# Dynamics of domain coverage of the protein sequence universe

**DOI:** 10.1186/1471-2164-13-634

**Published:** 2012-11-16

**Authors:** Bhanu Rekapalli, Kristin Wuichet, Gregory D Peterson, Igor B Zhulin

**Affiliations:** 1 Joint Institute for Computational Sciences, Oak Ridge National Laboratory – University of Tennessee, Oak Ridge, TN, 37831, USA; 2 Department of Microbiology, University of Tennessee, Knoxville, TN, 37996, USA; 3 Department of Electrical Engineering and Computer Science, University of Tennessee, Knoxville, TN, 37996, USA; 4 Present address: Max-Planck-Institute for Terrestrial Microbiology, Marburg, D-35043, Germany

## Abstract

**Background:**

The currently known protein sequence space consists of millions of sequences in public databases and is rapidly expanding. Assigning sequences to families leads to a better understanding of protein function and the nature of the protein universe. However, a large portion of the current protein space remains unassigned and is referred to as its “dark matter”.

**Results:**

Here we suggest that true size of “dark matter” is much larger than stated by current definitions. We propose an approach to reducing the size of “dark matter” by identifying and subtracting regions in protein sequences that are not likely to contain any domain.

**Conclusions:**

Recent improvements in computational domain modeling result in a decrease, albeit slowly, in the relative size of “dark matter”; however, its absolute size increases substantially with the growth of sequence data.

## Background

The protein universe is the collection of all proteins of every biological species that lives or has lived on earth [1]. Its basic properties are the subject of rigorous investigation [2,3], because it is an essential foundation of all biology. The currently known protein space, which is a part of the protein universe that has been revealed by DNA sequencing, consists of more than 16 million protein sequences in a non-redundant (nr) database (December 8, 2011) and its size is rapidly increasing due to recent technological advances [4,5]. Only a small fraction of the current protein space can be analyzed by traditional experimental techniques therefore, computational classification of protein sequences and their assignment to known biological functions is critical [6,7].

Proteins are composed of one or more domains, parts that are conserved in sequence and structure and that can evolve and function independently [8]. Several valid and often overlapping definitions of protein domains exist, starting with the original definition by Wetlaufer, as stable units of protein structure that could fold autonomously [9]. In terms of protein sequences, domains are clusters of consecutive residues exhibiting various levels of conservation. Domains vary in length between 40 to nearly 700 residues [10]; however, 90% of surveyed domains are shorter than 200 residues [11] with an average of approximately 100 residues [12].

The use of profile hidden Markov models (HMMs) that capture the conserved sequence features of protein domains [7,13,14] is arguably the most successful computational approach for identifying protein domains, and the Pfam (Protein Families) database is the premier repository, currently containing 13,672 protein domain models in its high-quality, curated Pfam-A part [15]. Another popular resource, a Conserved Domain Database (CDD) at the National Center for Biotechnology Information [16], is a larger, partially redundant collection of domain and multi-domain models imported from various sources, including Pfam. ProDom [17] and ADDA [18] are also important resources aiming at developing high-quality domain models. Using Pfam and CDD profiles, recent computational analyses have assigned 72% of all protein sequences in the NR database [1] and nearly 80% of all sequences in the curated UniProtKB database [15] to known protein families. The remaining sequences are uncharacterized and considered to be “dark matter” of the protein universe [1]. Levitt [1] proposed four potential components comprising “dark matter”: (i) sequences that are erroneous; (ii) low-complexity, non-globular sequences; (iii) known but unrecognized protein domains; and (iv) novel protein domains to be discovered.

In this study, we propose to expand the definition of “dark matter” by including regions in partly covered protein sequences that are not characterized and do not have any domain match. In addition to domain coverage, detecting regions in protein sequences that are unlikely to contain any domain considerably reduces the size of “dark matter”. Finally, we show that despite substantial improvements in computational domain modeling and tools for their identification, the relative size of “dark matter” decreases slowly, while its absolute size increases dramatically with the growth of sequence data.

## Results and discussion

### Further defining “dark matter” of the protein sequence universe

**Figure 1:**
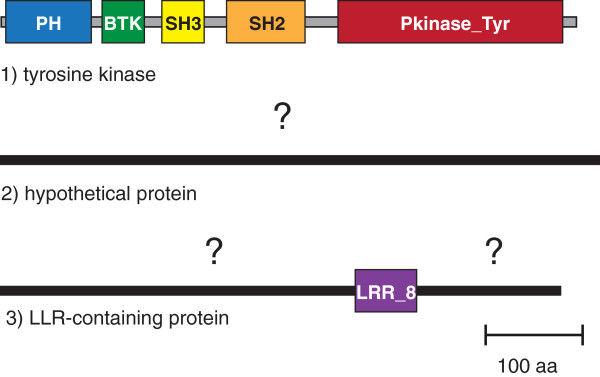
**Different levels of computational coverage in protein sequences.** Three representative proteins from the human genome are shown: (1) a tyrosine kinase (GI: 307508) has a comprehensive coverage by five Pfam domains (shown as colored rectangles with their respective names). Sequence regions that are less than 50 aa long are shown as grey lines; (2) a hypothetical protein (GI: 341913853) has no matches to any known protein domain or region and is considered part of “dark matter” (shown as a black line with a question mark above); (3) a leucine-rich repeat-containing protein is characterized only partly by a match to the LLR_8 (leucine-rich repeat) domain; however two large portions of its sequence (90% of total amino acid residues) show no matches to any domain or region, and therefore should be considered a part of “dark matter” (black lines with question marks above).

Currently defined “dark matter” of the protein sequence universe includes protein sequences that cannot be matched to any known protein family [1]. This definition does not seem to include a vast amount of unknown protein space. Many sequences have one or more matches to known protein domains, but still contain long stretches that are not computationally characterized. To illustrate this point, let us consider three protein sequences of a similar length from the human genome (Figure 1). A tyrosine kinase protein has a comprehensive (83%) coverage by five Pfam domains. The remaining 17% of sequence length is occupied by six short (4 to 40 aa) interdomain regions that are unlikely to contain any domain. This is an example of a perfect computational coverage from which a biological function can be deduced. An opposing case is the hypothetical protein, which has no matches to any domain. This sequence clearly belongs to “dark matter”. The third example shows a leucine-rich repeat (LRR) containing protein. According to a current definition [1], it is not considered as a part of “dark matter”, because it has a match to the Pfam LRR profile. However, 90% of its sequence shows no matches to any protein domain or region: its large N-terminal and C-terminal regions remain unknown. This protein cannot be assigned to any protein family and even its general function cannot be predicted. The current protein sequence database contains hundreds of thousands of protein sequences with incomplete computational coverage. We propose that uncharacterized parts of protein sequences should also be considered “dark matter”, because they match its definition: they may contain novel domains, undetected domains, non-globular linkers or erroneous sequences.

Many resources for computational domain finding exist. The original “dark matter” analysis by Levitt utilized CDD profiles [1]. However, we argue that while CDD is superior in overall computational coverage, it may not be the best choice for specifically defining protein domains. Many CDD profiles are built from sources such as Clusters of Orthologous Groups of Proteins (COG) [19] and Protein Clusters (PRK) [20] that are not specialized domain databases (e.g., COG focuses on evolutionary relationships and PRK on basic relatedness between protein sequences). Both COG and PRK capture similarity between protein sequences regardless of their domain composition. As a result, many CDD profiles cover full-length proteins including regions for which domain information is unavailable. In contrast, the Pfam models are built primarily for protein domains and are known for excellent specificity. As such, Pfam models are integrated in many other resources including CDD.

**Figure 2:**
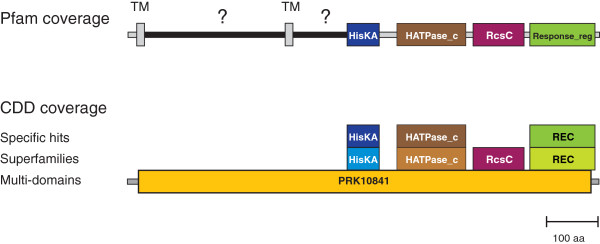
**Example of Pfam and CDD coverage of a protein sequence.** A protein sequence RcsC from *Escherichia coli* (GI: 145698285) is covered by four Pfam domain profiles: HisKA, HATPase_c, RcsC and Response_reg. Two transmembrane regions (TM) identified in this sequence by the TMHMM program are shown as grey rectangles. Small (<50 a.a.) interdomain regions are shown as grey lines. Large (>50 a.a.) interdomain regions are shown as black lines with a question mark. CDD profiles constructed from corresponding Pfam and SMART [14] domain models are confirmatory (redundant) and the only new information is provided by one additional profile, PRK10841, which covers the entire sequence.

This point can be further illustrated by the following example. Figure 2 shows computational coverage by Pfam and CDD profiles of a well-studied protein, the RcsC histidine kinase from *Escherichia coli*[21]. Fifty percent of the RcsC sequence is covered by four Pfam domains (HisKA, HATPase_c, RcsC and Response_reg). The CDD coverage of the same sequence is largely confirmatory: four CDD profiles are redundant (i.e., they correspond to regions that have been identified by the four Pfam profiles). The only new profile, which matches parts of the sequence that were not covered by Pfam profiles, is PRK10841. This profile captures sequence similarity between a dozen or so proteins from closely related to *E. coli* enterobacterial species, a fact that has been also well established in the literature [19]. The coverage by PRK10841 is important. It implies that throughout the entire length of this sequence a certain degree of similarity with other, independently obtained sequences is observed. This essentially rules out a possibility that the N-terminal region of this sequence, which has no Pfam matches, is erroneous. However, it still remains unknown whether the large N-terminal region that is not covered by Pfam profiles (amino acid residues from 1 to 460) contains any known or novel domains or consists of non-globular linker-like segments. Thus, while this region is considered computationally covered, *de facto* it remains a part of “dark matter”.

Based on arguments presented above, we determined that Pfam domain models are better suited for the purpose of defining the size of “dark matter” in the protein sequence space. Furthermore, the data on Pfam coverage of a large sequence space is available for comparison. The latest Pfam release (Pfam 26) is reported to cover nearly 80% of protein sequences in the UniProtKB database, but only 57% of amino acid (aa) residues in all protein sequences in this database [15]. We ran Pfam 26 on the latest release of the NCBI nr database and found that it covers only 51.39% of amino acid residues in its 16.39 million sequences. Thus, the size of “dark matter”, defined as a lack of domain information, appeared to be nearly half of the currently known protein space. The difference between Pfam domain coverage of the UniProtKB reported by the Pfam team [15] and of the nr database reported here appeared to be significant. It may reflect the fact that UniProkKB is slightly smaller in size than the nr database, but it could also be due to potential problems in the way calculations are done on such a large data set. Access to original data is limited due to its prohibitive size (a flat file size is cumulatively over 600 MB); thus, it seems important to report numbers obtained in an independent analysis, especially because according to our calculations the size of “dark matter” is larger. To clarify this point, we have repeated our analysis using the latest release of the UniProkKB database (September 2012) and obtained 53.8% domain coverage, which is close to numbers reported by the Pfam team.

### Can identification of specific regions other than domains reduce the size of “ dark matter” ?

Parts of protein sequences that do not contain domains often contain smaller functional elements, such as transmembrane helices and signal peptides [22]. Low complexity regions [23], including coiled coils [24], are often found in the interdomain regions in protein sequences and are used for identifying domain boundaries [25]. If these elements are unlikely to be a part of any domain, then identifying them in and subtracting from “dark matter” may decrease its size substantially. Recent analysis revealed that transmembrane regions differ in their level of complexity and can be found both within and outside current domain models [26]; however, exact distribution of these regions within and between domains of the current protein space remains unknown. By matching all protein sequences in the nr database to the above-mentioned regions (see Methods), we have determined that they occupy approximately 16% of the currently known protein space (Table 1). On the other hand, more than half of this space is within protein domains. Furthermore, our results show that none of the four types of regions can be overwhelmingly found outside domain boundaries (Table 1). These results are somewhat surprising. While some transmembrane regions were expected to be located in protein domains (some Pfam domains consist of transmembrane regions entirely; for example, the GPCR superfamily, accession PF00001), more than 2/3 of them are located outside known domains. On the contrary, more than half of low-complexity regions, which were expected to be found predominantly between domains, are located within domain boundaries (Table 1). Thus, we cannot confidently subtract any type of protein regions from “dark matter” when searching for novel and unidentified protein domains.

**Table 1:** Computational coverage of the protein sequence space

**Sequence space**^**a**^		**All proteins**	**Protein regions**				**All regions**
			**LC**	**TM**	**CC**	**SP**	
Total sequence space	aa	5.64E + 09	4.14E + 08	3.74E + 08	6.78E + 07	5.43E + 07	9.10E + 08
	%	100	7.3	6.6	1.2	1.0	16.1
Domain space	aa	2.90E + 09	2.72E + 08	1.20E + 08	4.65E + 07	4.62E + 07	4.84E + 08
	%	51.4	9.4^b^	4.1^b^	1.6^b^	1.6^b^	16.7^b^

^a^Data for nr December 2011 is shown. Abbreviations: LC, regions of low complexity; TM, transmembrane regions; CC, coiled coils; SP, signal peptides.

^b^Shown as relative percentage with respect to the 51.4% of domain space.

### A large section of protein space can be safely subtracted from “ dark matter”

As we have shown above, various computationally identifiable regions in protein sequences (e.g. transmembrane helices, low-complexity regions, etc) cannot be used to reduce the size of “dark matter”. However, a large section of “dark matter” apparently can be effectively predicted not to contain any domain. Once all domains are identified in all protein sequences, we can identify regions that are both (i) too short to contain a domain and (ii) are located in positions between pairs of known domains or between a known domain and the protein terminus (N or C). For example, such positions are shown in grey on Figure 1. To calculate the contribution of such regions to the total sequence space, we decided to set their size limit at 50 aa. The reason behind this number is that whereas some domains are smaller than 50 aa, domains are never located adjacent to each other without at a least a small connecting linker. The average size of interdomain linkers was calculated to be 6-8 aa [27]. Thus, a 50 aa cutoff accounts for the smallest domains bordered by average-size linkers. We have calculated that such regions cover approximately 9% of the total protein sequence space (5.09E + 08 aa), which is quite significant. Thus, by subtracting these regions from current “dark matter”, we effectively decrease its size from 48.6% to 39.6%.

### Relative size of “ dark matter” is shrinking, albeit slowly

**Figure 3:**
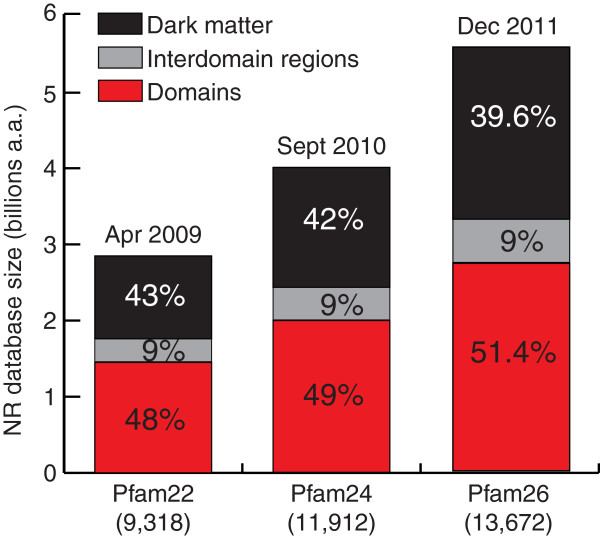
**Computational domain coverage of the protein sequence space from 2009 to 2011.** From April 2009 to December 2011, the NR database grew twice: from 2.8 to 5.6 billion aa. Three Pfam releases represent both model improvements and an increase in the number of domain models (shown in parentheses).

To find out how progress in sequencing and improvements in domain models change computational coverage of protein space, we have reconstructed past events by applying domain coverage by three consecutive Pfam releases: Pfam 22 [28], Pfam 24 [29] and Pfam 26 [15] to three releases of the NCBI nr database in years 2009, 2010, and 2011 (Figure 3) that correspond to each of the Pfam releases. Each new Pfam release constitutes not only an increase in the number of protein families covered (9,318 for Pfam 22; 11,912 for Pfam 24; and 13,672 for Pfam 26), but also significant improvements in domain models aiming at more comprehensive coverage while maintaining high specificity. We observed a 1.2% increase in domain coverage by Pfam 24 and an additional 2.5% increase by Pfam 26. Pfam developers report a 4% increase in coverage of the protein sequence space by Pfam 26 [15]. The difference again can come from the size of corresponding databases (nr and UniProtKB) or from calculations.

The trend shown on Figure 3 suggests that “the dark matter problem” is slowly being solved. The most recent advances in computational domain modeling and identification, such as the latest Pfam 26 release [15] and the underlying tool development [30], resulted in doubling the rate of improvement in domain coverage. However, the absolute size of “dark matter” is still growing rapidly as the genome sequencing progresses.

## Conclusions

Computational coverage of the protein sequence space, which is generated by genome sequencing projects, is an important process for our understanding of life. We propose a biologist-centered view on current computational coverage, where not only completely non-covered protein sequences, but also parts of partially covered protein sequences that are not occupied by protein domains are considered “dark matter”. Using high-throughput computing we show that the unexplored space of the protein sequence universe is larger than previously defined and that despite substantial improvements in bioinformatics during the last three years, the relative size of “dark matter” is decreasing very slowly.

## Methods

### Data sources

The following releases of the NCBI nr (non-redundant) database were used: April 4, 2009 (nrApr09), September 9, 2010 (nrSep10), and December 8, 2011 (nrDec11). The UniProtKB release September 2012 was used to calculate its domain coverage. Domain models/HMMs were retrieved from the three recent versions of the Pfam protein families database (Pfam-A portion only): Pfam 22.0 [28] Pfam 24.0 [29], and Pfam 26.0 [15]. Conserve Domain Database version 3.02 [16] was used to obtain its more than 78,000 position-specific scoring matrices (PSSMs).

### Software for identification of domains and regions in protein sequences

Protein sequence regions were identified using standard software packages and cutoffs: low-complexity regions, SEG [23]; coiled coils, PairCoil2 [31]; transmembrane regions, TMHMM2.0c [32]; and signal peptides, Phobius [33]. Protein sequences were scanned against Pfam domain models (profile HMMs) using *hmmscan* of the HMMER v.3.0 package [30] with the cut_ga filter and against CDD PSSMs using the RPS-BLAST [34] with default parameters. To fully reproduce earlier steps in computational domain coverage with Pfam 22.0 we used *hmmpfam* of HMMER v.2.3.2 adapted for the Kraken supercomputer, as described earlier [35]. The amino acid coverage was calculated for each protein sequence in the respective database based on the following considerations. For non-overlapping domains and regions the amino acid coverage is the sum of domain and region lengths. If a domain and a region overlap, the priority is given to the domain when computing domain coverage. For overlapping domains with satisfactory E values (above the threshold for domain identification), the length of the longest domain was taken into consideration.

### Computer environment

All computational analyses were performed in a local computing environment. Computationally intensive tasks were carried out using the Intel X86_64 Linux cluster (Newton) with a total of 4,200 processor cores at the University of Tennessee and the Cray XT5 supercomputer (Kraken) with a total of 112,896 processor cores at the Oak Ridge National Laboratory. Tasks were automated using a combination of C, PHP, and MPI scripts.

## Competing interests

The author(s) declare that they have no competing interests

## Authors' contributions

BR, GDP and IBZ conceived the study and designed the research. BR and GDP carried out software design and computational analysis. BR, KW and IBZ analyzed the results and wrote the paper. All authors read and approved the final manuscript.
